# Experiments on Various Subjects in Physiology

**Published:** 1820-05

**Authors:** 


FOR THE LONDON MEDICAL AND PHYSICAL JOURNAL.

### *Experiments on various Subjects in Physiology.*

IT is a peculiar mark of the wisdom and discrimination of these times, that the cultivation of the sciences proceeds solely upon the basis of experimental certainty. It was once the custom for men of talents only to engage themselves in this work; but it has been wisely determined, according to the newest inventions, that the increase of our knowledge has no dependence upon the intellectual qualifications of those who are occupied in its pursuits; that *facts*, and *facts only*, are to be sought after; and, provided *facts are increased*, the cause of science cannot choose but be promoted. This discovery is one of the most fortunate ever made for the benefit of mankind; for the greatest fools might multiply the acquisition of facts *in infinitum;* and, as this is a description of persons by no means rare, the career of scientific attainment will proceed, happily, not in a ratio to the genius employed upon it, but in proportion to the *number* of those whose services are engaged. Availing myself of the privilege of labouring with my contemporaries in those ample fields of science which are thus laid open, I request the favour of your giving publicity to the following experiments:

*Experiment* 1*st.*—I procured a dog, and had all his teeth drawn: I kept the dog twenty-four hours without food, and at that time gave him a bone; it was the tibia of an ox. The dog being hungry, applied his mouth to the bone, but could by no means eat it.

*Exp.* 2*d.*—The dog being kept twelve hours longer without food, had then offered to him a decoction of mutton, with some meat in it, and I think an onion: the temperature of the fluid was 136. The dog made many attempts to eat, but could not succeed. Half-a-pint of cold milk was then added, which reduced the temperature of the fluid to 96: the dog now ate greedily.

*Exp.* 3*d.*—Being desirous of illustrating certain physiological questions of considerable importance, I devised the following experiment, the subject of which was the above-mentioned dog. I directed that four inches of his tail should be completely shaved: this being done, the skin appeared of a white and delicate complexion. I then had the signs of the zodiac drawn on this shaved portion of his tail, in Indian ink, by an eminent artist; a small hole was drilled in his nose, through which I introduced a primrose; and an incision three inches in length was at the same time made in his back, which was filled with gunpowder; a large quantity of hobnails, with a few shavings, and ten grains of Cayenne pepper, having been previously introduced into the stomach. I now directed my assistants at once to cut off the tail of the dog, to pull out the primrose, and to fire the gunpowder. This was suddenly and adroitly performed by my friends, Drs. Split, Cut, and Green. The dog appeared to be very much astonished, but did not speak a word. Ten minutes afterwards the dog was killed, and the stomach examined. The general appearances of the stomach were natural, except that there was a slight redness observable about the cardiac orifice: a considerable secretion had taken place, and the Cayenne pepper appeared to have undergone a complete solution. There was, however, no trace of a digestive operation either upon the shavings or the hobnails.

*Exp. 4th.*—I procured a large cock, and plucked three feathers from his right wing, and five from his left; two feathers were also extracted from his tail. The cock being then placed on the ground, flapped his wings, and crowed twice; and, on some barley being offered to him, he afterwards began to eat.

*Exp. 5th.*—I procured a rabbit, and divided several of his nerves. I then subjected him to the action of a galvanic battery, consisting of 326 twelve-inch plates, directing refrigeration to be at the same time maintained by means of a pair of bellows. It was observed by some of the spectators that he made some attempts at wagging his tail; and it was thought that he would have laughed, if he could. The rabbit was then boiled in the usual way.

I beg that, in your next Proëmium, the above experiments may receive a proper notice. It may not be uninteresting to naturalists to be informed, that I now have two specimens of the crania of monkeys: that the chin (I am positive 'tis a chin) of one of them measures half-an-inch, and that of the other is about a quarter of an inch shorter. One of these monkeys has a rib four inches in length, which forms an unusually acute angle; and they both of them once had tails. These facts, which are highly important, may not be unworthy the notice of some of our zoologists.

N. B. It was a severe frost on the night of the 3d of January.

L. G. *a retired Captain of Mutilators.*

*March 12th,* 1820.

